# A Linkage between SmeIJK Efflux Pump, Cell Envelope Integrity, and σ^E^-Mediated Envelope Stress Response in *Stenotrophomonas maltophilia*

**DOI:** 10.1371/journal.pone.0111784

**Published:** 2014-11-12

**Authors:** Yi-Wei Huang, Rung-Shiuan Liou, Yi-Tsung Lin, Hsin-Hui Huang, Tsuey-Ching Yang

**Affiliations:** 1 Department of Biotechnology and Laboratory Science in Medicine, National Yang-Ming University, Taipei, Taiwan; 2 Division of Infectious Diseases, Department of Medicine, Taipei Veterans General Hospital, Taipei, Taiwan; 3 School of Medicine, National Yang-Ming University, Taipei, Taiwan; Arizona State University, United States of America

## Abstract

Resistance nodulation division (RND) efflux pumps, such as the SmeIJK pump of *Stenotrophomonas maltophilia*, are known to contribute to the multidrug resistance in Gram-negative bacteria. However, some RND pumps are constitutively expressed even though no antimicrobial stresses occur, implying that there should be some physical implications for these RND pumps. In this study, the role of SmeIJK in antimicrobials resistance, envelope integrity, and σ^E^-mediated envelope stress response (ESR) of *S. maltophilia* was assessed. SmeIJK was involved in the intrinsic resistance of *S. maltophilia* KJ to aminoglycosides and leucomycin. Compared with the wild-type KJ, the *smeIJK* deletion mutant exhibited growth retardation in the MH medium, an increased sensitivity to membrane-damaging agents (MDAs), as well as activation of an σ^E^-mediated ESR. Moreover, the expression of *smeIJK* was further induced by sub-lethal concentrations of MDAs or surfactants in an σ^E^-dependent manner. These data collectively suggested an alternative physiological role of *smeIJK* in cell envelope integrity maintenance and σ^E^-mediated ESR beyond the efflux of antibiotics. Because of the necessity of the physiological role of SmeIJK in protecting *S. maltophilia* from the envelope stress, *smeIJK* is constitutively expressed, which, in turn, contributes the intrinsic resistance to aminoglycoside and leucomycin. This is the first demonstration of the linkage among RND-type efflux pump, cell envelope integrity, and σ^E^-mediated ESR in *S. maltophilia*.

## Introduction

Efflux pump systems are present in all organisms and recognized to transport a range of structurally unrelated compounds and confer clinically relevant resistances to antimicrobial agents. There are five families of efflux pumps associated with multidrug resistance (MDR): the ATP binding cassette (ABC) family, the multidrug and toxic-compound extrusion (MATE) family, the major facilitator (MFS) family, the resistance nodulation division (RND) family, and the small multidrug resistance (SMR) family [1]. The RND efflux pumps consist of a RND-type transporter protein, which is located in the inner membrane; a membrane-fusion protein (MFP), which is located in the periplasmic space; and an outer membrane protein, which is located in the outer membrane of the bacterium. Many studies have evidenced that the RND-type efflux pumps not only confer resistance to drugs but also have a physiological role in stress adaption [2]. It is possible that the efflux pump-mediated antibiotics resistance is a by-product of the physiological role(s) of these pumps, especially for the constitutively expressed efflux pumps. Several proposed examples can evidence this viewpoint; for example, the AcrAB-TolC system of *Escherichia coli* and the CmeABC system of *Campylobacter jejuni* can export noxious metabolites, such as bile acid, produced by the host organism [3], [4]. In addition, several RND-type efflux pumps display inducibly expression upon the treatment of stresses, further linking the stress adaption and the efflux pumps.

Bacteria encounter an array of potentially growth compromising conditions in nature. Cell envelope is the major component of the defense against environmental threats for bacteria. Bacterial cells possess a variety of mechanisms to monitor and keep the cell envelope integrity. A variety of stresses, which affect components of the cell envelope, will intrigue the envelope stress responses (ESRs) [5]. In response to the various extracellular changes, extracytoplasmic function (ECF) σ factor provides a mean to sense external changes and regulate gene expression to prevent or repair cellular damages caused by stresses [6]. One of the best-studied ECF σ factors is the σ^E^, which is a key regulator of ESRs in *E. coli* and other Gram-negative bacteria [7]. In unstressed cells, σ^E^ generally interacts with an antisigma factor RseA, a single-pass inner membrane protein, that prevents σ^E^ from interacting with RNA polymerase and keeps σ^E^ inactive. When envelope stresses occur, inhibition of σ^E^ is relieved by the complete degradation of RseA via regaulted intramembrane proteolysis (RIP) [8]. The activated σ^E^ acts as a transcription factor to affect the expression of σ^E^ regulon genes. In most cases, the *rpoE* and *rseA* genes are organized in an operon.

*Stenotrophomonas maltophilia*, ubiquitous in environment, is an opportunistic pathogen involved in many nosocomial infections [9]. The genome information of *S. maltophilia* K279a predicted that there are eight putative RND efflux pumps, SmeABC, SmeDEF, SmeGH, SmeIJK, SmeMN, SmeOP, SmeVWX, and SmeYZ [10]. Of them, the SmeIJK pump is special because it consists of two different RND-type transporters, SmeJ and SmeK. It has been reported that the *smeIJK* operon is intrinsically expressed and can be further overexpressed in some mutants. The expression of *smeIJK* confers the resistance to aminoglycosides, tetracycline, minocycline, ciprofloxacine, and levofloxacin [11]. However, the constitutive expression of the *smeIJK* operon in strains maintained in the absence of antibiotic selective pressure raises the possibility that drug extrusion is not the only or main function of the SmeIJK pump. Accordingly, the physiological function of *smeIJK* was further assessed in this study.

## Materials and Methods

### Bacterial strains and culture conditions

A complete list of strains, plasmids, and primers used in this study is shown in Table S1.

### Construction of deletion mutants

Four PCR amplicons (labeled as I-IV in Fig. S1) were amplified using primer sets of SmeI5-F/SmeI5-R, SmeJ5-F/SmeJ5-R, SmeK5-F/SmeK5-R, and SmeK3-F/SmeK3-R (Table S1), respectively. Amplicons II and III were subsequently cloned into pEX18Tc to yield the recombinant plasmid pΔSmeJ, in which the cloned *smeJ* gene was partially deleted. Similar constructs for pΔSmeK and pΔSmeIJK were done by assembling the amplicons of III and IV as well as I and IV respectively, yielding pΔSmeK and pΔSmeIJK. Three PCR amplicons (labeled as I-III in Fig. S2) were amplified using primer sets of RpoE5-F/RpoE5-R, RpoE3-F/RpoE3-R, and RseA3-F/RseA3-R (Table S1), respectively. Recombinant plasmids pΔRpoE and pΔRseA were obtained by subsequently cloning the amplicons I and II as well as II and III into pEX18Tc. The plasmids mobilization, transconjugants selection, and mutant confirmation were performed as described previously [12]. The *smeJK* and *rpoErseA* double mutants were constructed from a single mutant.

### Construction of the *rpoE* expression plasmid, pRpoE

The intact *rpoE* gene was PCR amplified from genomic DNA template of *S. maltophilia* KJ by using primer sets of RpoE5-F and RpoE3-R and then cloned into plasmid pRK415, generating plasmid pRpoE.

### Susceptibility testing

The antibacterial activities of agents were determined on Mueller-Hinton agar plates. Agar plates were prepared by the twofold agar dilution technique recommended by Clinical and Laboratory Standards Institute (CLSI) [13]. The MIC was defined as the lowest concentrations that inhibited bacterial cell growth. All antimicrobial agents used were purchased from Sigma Aldrich.

### In vitro growth curves

Overnight cultures of *S. maltophilia* were inoculated into the fresh medium to the *A*_450_ of 0.15. Cells were grown aerobically and the *A*_450_ was measured every 3 h.

### Osmotic challenge assay

The 24-h cultured bacterial cells of strains KJ, KJΔIJK, KJΔJ, and KJΔK were inoculated into fresh LB or MH broths containing different concentrations of NaCl with an *A_450 nm_* of 0.15. The bacteria were further cultured for 5 h and the *A_450 nm_* were recorded. The relative survival percentage of individual mutant to the wild-type KJ, at each cultured condition, was calculated. These experiments were performed at least three times.

### Sodium dodecyl sulfate (SDS) survival analysis

Overnight cultures of the tested strains were diluted to *A*_450 nm_ of 0.15 with LB or MH broth. Cells grown to stationary phase were adjusted to *A*_450 nm_ of 1.0 with the same broth. The cells were treated with or without 0.02% SDS. A final *A*_450 nm_ measurement was taken after 10 min of incubation without shaking. 100% survival was defined as the absorbance of each strain without SDS. The percentage of survival was defined as the *A*_450 nm_ ratio of the SDS-additive group to the SDS-free counterpart.

### Polymyxin E susceptibility test

LB or MH plates were streaked with a cotton swab soaked in *S. maltophilia* cell suspension of 10^7^ cells/ml. The commercial discs of 10 mg polymyxin E was placed at the centre onto the agar surface. The culture was then incubated at 37°C for 24 hours. The diameter of a zone of inhibition was measured (in millimeters). Each experiment was repeated at least three times.

### Construction of *P_smeI_-xylE* and *P_rpoE_-xylE* transcription fusion, pSmeI_xylE_ and pRpoE_xylE_

The 465-bp DNA fragment upstream of *smeIJK* operon and the 414-bp DNA fragment upstream of the *rpoE-rseA-mucD* operon were obtained by PCR using primer sets SmeI5-F/SmeI5-R (Fig. S1 & Table S1) and RpoE5-F/RpoE5-R (Fig. S2 & Table S1), respectively. The plasmid-borne transcription fusions, pSmeI_xylE_ and pRpoE_xylE_, were constructed by ligating the 465-and 414-bp DNA fragment into the *xylE-*reporter plasmid pRKXylE, respectively. The plasmids, pSmeI_xylE_ and pRpoE_xylE_, were mobilized into *S. maltophilia* strains indicated for promoter activity assay.

### Stress challenge assays

Overnight cultures of KJ(pSmeI_xylE_) were subcultured into fresh LB medium with an*A_450 nm_* of 0.15. After 5 h incubation (37°C, 170 rpm), the KJ(pSmeI_xylE_) cells were divided into two parts. One part served as a non-treated control and the second part received different stress challenges for 3 h. The stress included Triton X-100 (100 µg/ml), benzalkonium chloride (BC) (10 µg/ml), cetyltributylammonium bromide (CTAB) (10 µg/ml), gentamicin (5 µg/ml), amikacin (5 µg/ml), and leucomycin (1 µg/ml). All antimicrobial agents used were purchased from Sigma Aldrich. The C23O activity of KJ(pSmeI_xylE_) was determined. The C23O activity determined from the non-treated control was regarded as 100%.

### Catechol 2,3-dioxygenase (C23O) activity assay

Catechol-2,3-dioxygenase is encoded by the *xylE* gene and its activity was measured as the rate of increase in *A_375 nm_* following the addition of 100 mM catechol, as described elsewhere [14]. The rate of hydrolysis was calculated by using 44,000 M^−1^cm^−1^ as the extinction coefficient. One unit of enzyme activity (U) was defined as the amount of enzyme that converts 1 nmole substrate per minute. The specific activity was expressed as U/OD_450 nm_.

## Results

### Either SmeJ or SmeK protein can support the SmeIJK pump function

Eight RND-type efflux systems were predicted to be present in the *S. maltophilia* K279a genome [10]. Of them, the *smeIJK* is unique for the presence of two tandem RND-type inner membrane transporters (Fig. S1). The SmeI protein, encoded by the annotated Smlt4279 gene, was predicted to be an MFP, as well as SmeJ and SmeK, encoded by the annotated Smlt4280 and Smlt4281 genes respectively, to be RND-type inner membrane transporters. SmeJ and SmeK showed a significant similarity (42% identity and 59% similarity). To assess whether both RND transporters are required for pump function or either RND transporter can independent associate with SmeI and cognate outer membrane protein for the assembly of functional tripartite pumps, we constructed strains with various combinations of deletion in RND transporters and assessed efflux pump function by the susceptibility test. KJΔJ, KJΔK, KJΔJK, and KJΔIJK were the *smeJ*, *smeK*, *smeJK*, and *smeIJK* isogenic mutants of wild-type KJ, respectively (Fig. S1). The consideration of polar effect in KJΔJ was assessed by qRT-PCR. The *smeK* transcript of KJΔJ was comparable to that of the wild-type KJ, indicating that inactivation of *smeJ* had no polar effect on the expression of downstream *smeK* gene. Eight- to 16-fold increase in the susceptibility to aminoglycoside and 4-fold decrease in the resistance to leucomycin were observed for KJΔJK, as compared to the MICs of the parent strain KJ. Deletion of *smeJ* or *smeK* alone slightly altered the susceptibility of strain KJ to the aminoglycosides tested (two-fold MIC difference) (Table 1). Although the acceptable inaccuracy for the susceptibility test is in the range of 2-fold MIC value, it cannot be immediately ruled out that SmeJ and SmeK may be partially functionally redundant and simultaneous inactivation of *smeJK* may has an additive effect on aminoglycosides extrusion. Furthermore, Table 1 also demonstrates that mutants KJΔJK and KJΔIJK displayed the same susceptibility to all the antimicrobials tested, supporting that *smeI* deletion makes little contribution to the additive phenotype of *smeJK* mutant.

**Table 1:** Antimicrobial susceptibilities of *S. maltophilia* KJ and its derived deletion mutants.

Antimicrobial	MIC (µg/ml)				
	KJ	KJΔJ	KJΔK	KJΔJK	KJΔIJK
**Chloramphenicol**	8	8	8	8	8
**Quinolone**					
Nalidixic acid	8	8	8	8	8
Norfloxacin	16	16	16	16	16
**Tetracycline**					
Deoxycycline	1	1	1	0.5	0.5
Tetracycline	16	16	16	8	8
**Aminoglycoside**					
Amikacin	1024	512	512	64	64
Gentamicin	1024	512	512	64	64
Kanamycin	256	128	128	32	32
Tobramycin	512	256	256	64	64
**Macrolide**					
Erythromycin	64	64	64	64	64
Leucomycin	256	256	256	64	64
Rokitamycin	512	512	512	512	512

### The *smeIJK* mutant displays a compromised growth in Mueller-Hinton (MH) medium, but not in Luria-Bertani (LB) medium

The phenotype of KJΔIJK grown in the MH agar attracted our attention during the susceptibility test. In comparison, bacterial lawn of KJΔIJK on the MH agar was homogeneously smaller and thinner than those of KJ, KJΔJ, and KJΔK. Nevertheless, the bacterial lawns of KJ, KJΔJ, KJΔK, and KJΔIJK were almost the same size in the LB agar.

**Figure 1:**
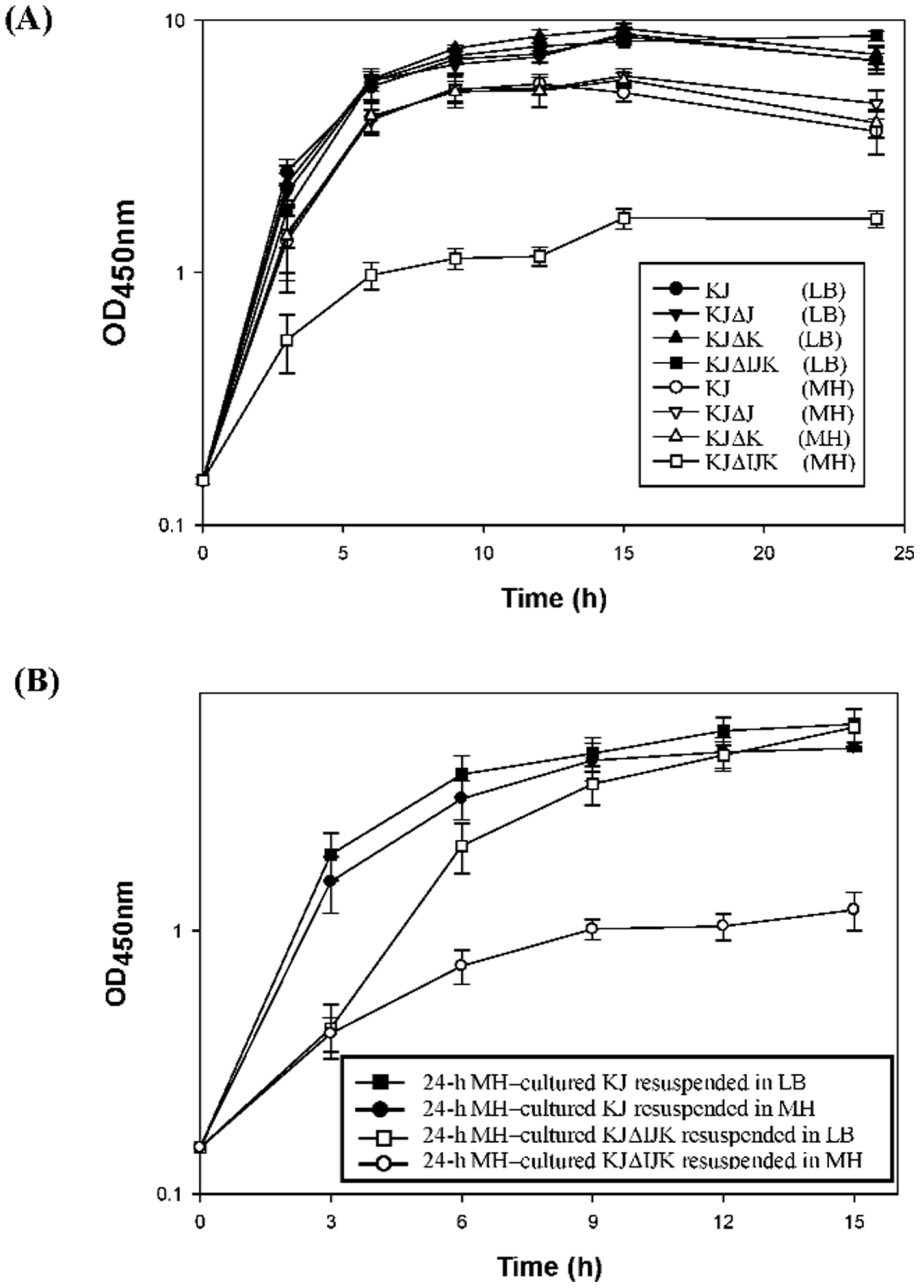
Growth of KJ, KJΔJ, KJΔK, and KJΔIJK in Luria-Bertani (LB) and Mueller-Hinton (MH) media. (A) Growth curves of KJ, KJΔJ, KJΔK, and KJΔIJK in LB and MH broth. (B) The growth curve of 24-h MH-cultured KJ and KJΔIJK cells subcultured into the MH or LB media.

To further clarify this observation, the growth curves of KJ, KJΔJ, KJΔK, and KJΔIJK in MH and LB broth were monitored. As shown in Fig. 1A, KJ, KJΔJ, and KJΔK grown in the MH broth exhibited slightly reduced growth rates compared to those grown in the LB broth. However, the growth patterns of KJΔIJK were distinctly different when it grew in MH and LB broth. In the MH broth, the doubling time of KJΔIJK was approximately 1.71-fold longer than that of KJ, KJΔJ, and KJΔK. In the meantime, the maximum OD_450_ achieved for KJΔIJK in the MH broth was approximately 1.63, which was significantly lower than those for KJ, KJΔJ, and KJΔK in the MH broth. Nevertheless, the growth curve for KJΔIJK grown in the LB broth was indistinguishable from those for KJ, KJΔJ, and KJΔK (Fig. 1A).

### The growth retardation of KJΔIJK in MH medium can be reverted to normal when the bacteria were shifted from MH medium into LB medium

Since RND-type efflux pump has been known to be responsible for extrusion of noxious compounds, we speculated that the growth retardation of *smeIJK* mutant in the MH broth may result from the accumulation of some noxious metabolites, which should be extruded by the SmeIJK pump. The putative noxious metabolites should be present in the MH broth, but absent in the LB broth. To test this assumption, the bacterial cells of KJ and KJΔIJK grown in the MH broth for 24 h were collected and resuspended into a fresh LB broth and MH broth, respectively, to an *A_450 nm_* of 0.15, and then the growth curves of KJΔIJK in the LB broth and MH broth were monitored. If there are noxious metabolites present in the 24-h MH-cultured KJΔIJK cells, the initial growth rate must be significantly retarded when the cells were shifted to the fresh MH broth. However, when they were shifted to the fresh MH medium, the 24-h MH-cultured-KJΔIJK cells still can logarithmically grow for 9 h (Fig. 1B). Furthermore, the growth of 24-h MH-cultured-KJΔIJK cells was gradually reverted to the wild-type pattern after LB broth shift (Fig. 1B). Therefore, the *ΔsmeIJK*-mediated growth retardation in the MH broth is reversible when the cultured environment is changed from the MH broth to the LB broth, suggesting that a component present in MH medium but absent from LB medium determined the growth compromise in MH medium.

### *SmeIJK* mutant displayed decreased tolerance to hypo-osmolarity and casein hydrolysate

**Figure 2:**
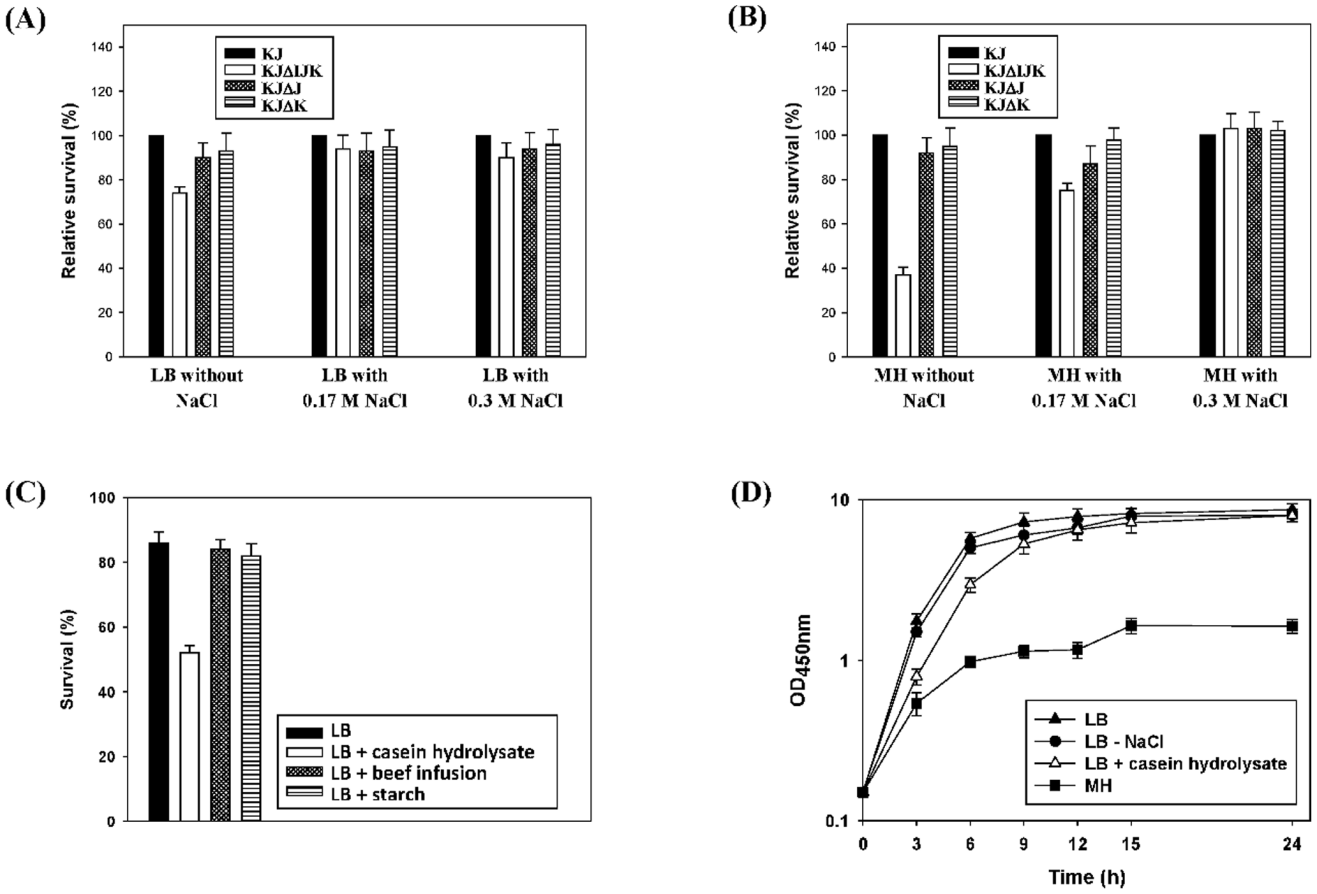
The role of SmeIJK pump in the tolerance to hypo-osomolarity and casein hydrolysate. The data are the average of the measurements made in triplicate. (A) The relative survival of KJΔIJK, KJΔJ, and KJΔK to the wild-type KJ in the LB medium containing different concentrations of NaCl. The relative survival percentage of individual mutant to the wild-type KJ, at each cultured condition, was calculated using the OD_450 nm_ of the wild-type KJ as 100%. (B) The relative survival of KJΔIJK, KJΔJ, and KJΔK to the wild-type KJ in the MH medium containing different concentrations of NaCl. The relative survival percentage of individual mutant to the wild-type KJ, at each cultured condition, was calculated using the OD_450 nm_ of the wild-type KJ as 100%. (C) The sensitivities of KJ to SDS in LB or LB containing casein hydrolysate, beef infusion, or starch were determined by the OD_450 nm_ measurement. The percentage of survival was defined as the OD_450 nm_ ratio of the SDS-additive group to the SDS-free counterpart. (D) Growth curves of KJΔIJK grown in the media of the LB, the LB without NaCl, the LB with casein hydrolysate, and the MH, respectively.

An MH medium typically contains 0.3% beef infusion, 1.75% casein hydrolysate, and 0.15% starch. The ingredients for LB media were 1% tryptone, 0.5% yeast extract, and 1% (0.17 M) NaCl. Given the compositions of the MH and LB media, we speculated that the observed growth retardation in the MH medium was due to the absence of NaCl in the medium. To test this assumption, three LB-based media with a final NaCl concentration of 0, 0.17, and 0.3 M were prepared. The osmotic challenge assay to KJ, KJΔJ, KJΔK, and KJΔIJK cells was determined. KJΔIJK displayed a reduced survival in the 0 M NaCl LB medium when compared to the wild-type KJ. Nevertheless, the survival of KJΔIJK cells restored to nearly wild-type levels in the LB supplemented with 0.17 M and 0.3 M NaCl (Fig. 2A). The phenotype correlated *smeIJK* to the hypo-osmolarity tolerance. Furthermore, the survivals of KJΔJ and KJΔK were similar to that of the wild-type KJ in spite of the concentration of NaCl in the cultured medium (Fig. 2A). The osmotic challenge assay of KJ, KJΔJ, KJΔK, and KJΔIJK was also performed in three MH-based media containing 0, 0.17, and 0.3 M NaCl. The results concluded from the MH counterpart (Fig. 2B) is consistent with those from the LB counterpart. It is worthily noted that the *ΔsmeIJK*-mediated compromise in hypo-osmolarity tolerance was more apparent in the MH counterpart than that in the LB counterpart, implying that some components, in addition to NaCl, in the MH medium may cause an envelope stress to *S. maltophilia*. To test this assumption, we supplemented LB with individual compounds present in MH medium to determine which component affected susceptibility to SDS. Only the addition of casein hydrolysate increased the sensitivity of KJ cells to SDS (Fig. 2C)

To further elucidate whether the presence of casein hydrolysate and the absence of NaCl result in the *ΔsmeIJK*-mediated growth compromise in the MH medium. The growth curves of KJΔIJK cells grown in the LB, the LB without NaCl, the LB with casein hydrolysate, and the MH were monitored. As shown in Fig. 2D, the logarithmic-phase growth of KJΔIJK cells was compromised when casein hydrolysate was added into the LB medium; however, the stationary-phase growth of KJΔIJK cells in the LB with casein hydrolysate was comparable to that in the LB.

### Loss of SmeIJK compromised the cell envelope integrity

**Figure 3:**
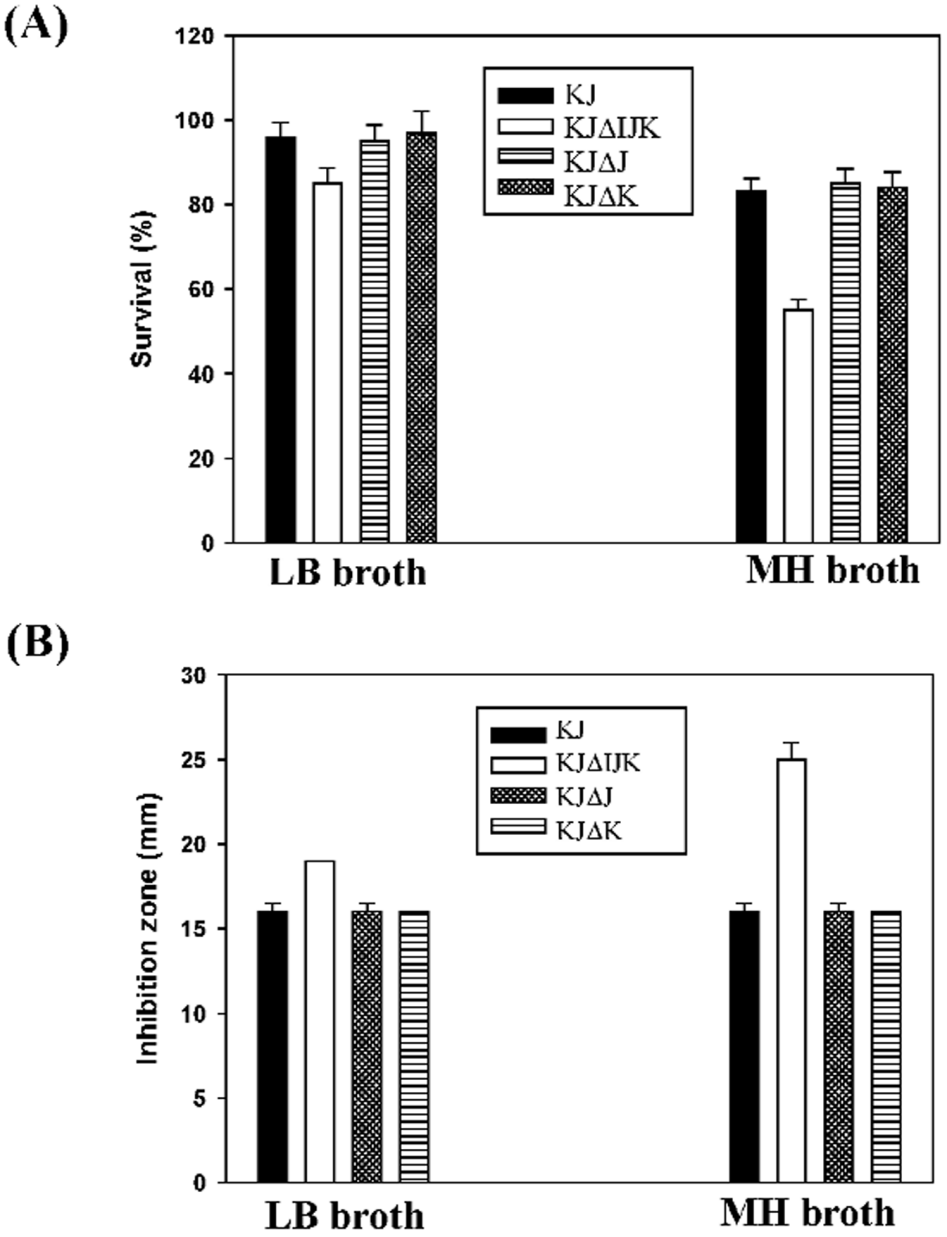
Assessment of the cell envelope integrity of *S. maltophilia* KJ and its derived mutants in different cultured conditions. (A) Sodium dodecyl sulfate (SDS) survival analysis. The survival of KJ, KJΔIJK, KJΔJ, and KJΔK in LB or MH broth without or with 0.02% SDS was determined by the *A_450 nm_* measurement. The percentage of survival was defined as the *A_450 nm_* ratio of the SDS-additive group to the SDS-free counterpart. (B) Polymyxin E susceptibility. The polymyxin E susceptibility of KJ, KJΔIJK, KJΔJ, and KJΔK in LB or MH broth was determined by the disc diffusion assay.

Compared with the wild-type KJ, KJΔIJK was more sensitive to hypo-osmolarity environments (Fig. 2). KJΔIJK can thus exhibit a perturbation in the cell envelope integrity. We performed the survival experiments with a 0.02% SDS challenge for 10 min on cells. KJΔIJK was found to be more susceptible to SDS stress than the parental strain KJ. Moreover, the LB-grown KJΔIJK cells were more tolerant to SDS challenge than the MH-grown ones (Figure 3A). KJΔJ and KJΔK cells kept the similar SDS sensitivity as the wild-type KJ cells did.

Polymyxins can bind to lipopolysaccharide (LPS) in the outer membrane of Gram-negative bacteria, disrupting both the outer and inner membranes. Therefore, the susceptibility to polymyxins should be increased if the cell envelope integrity of bacteria is compromised. The polymyxin E susceptibility of KJ, KJΔIJK, KJΔJ, and KJΔK was thus assessed. KJΔIJK was more sensitivity to polymyxin E than KJ, KJΔJ, and KJΔK, especially in the MH medium (Fig. 3B).

### Loss of SmeIJK activated the σ^E^ regulon

**Figure 4:**
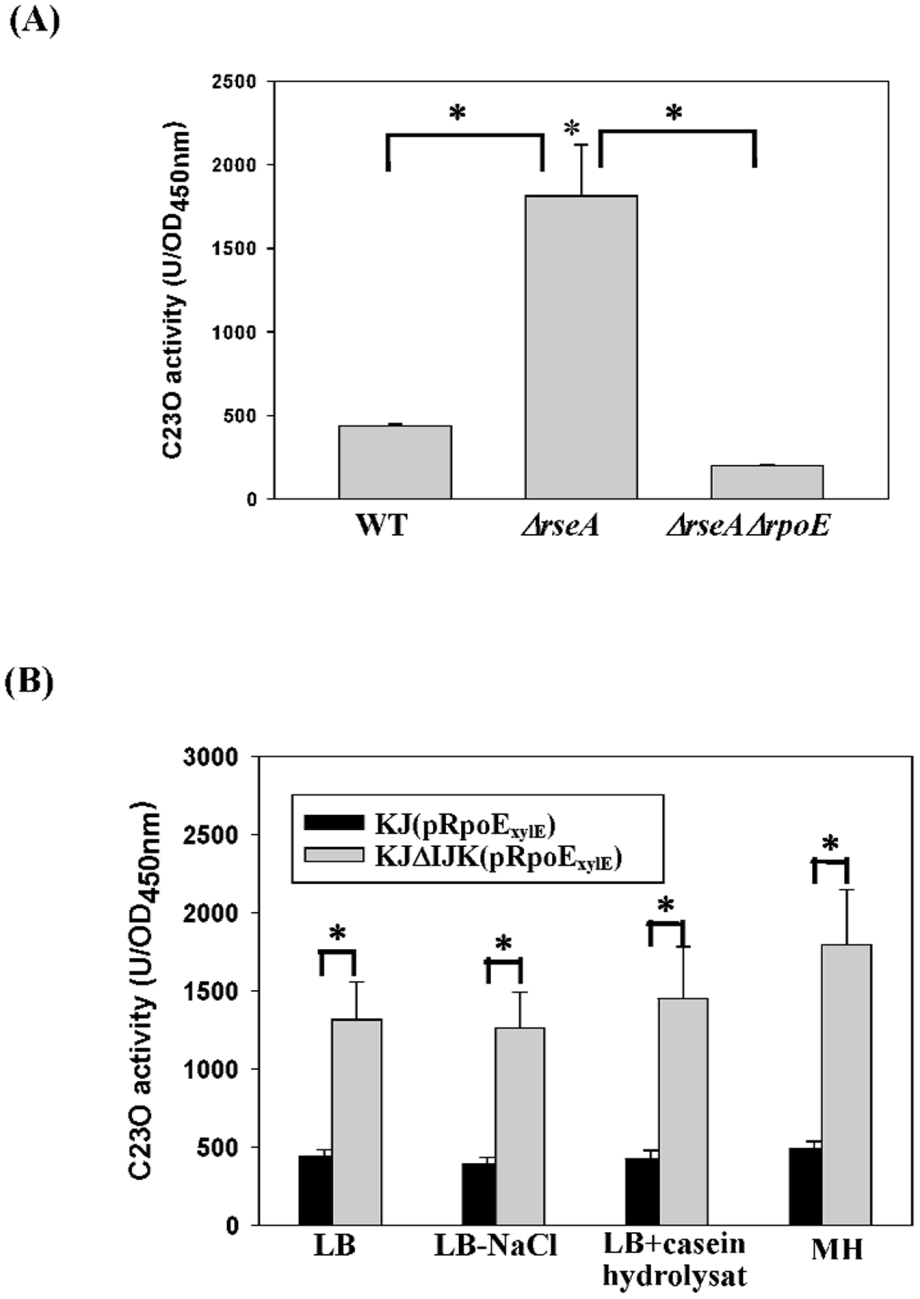
Determination of *P_rpoE_* activities in different strains and culture conditions. Plasmid containing a transcriptional fusion of the upstream region of *rpoE* to the *xylE* gene (pRpoE_xylE_) was transferred into the wild-type KJ and its derived mutants. The C23O activities of the logarithmic-phase cultures of these strains were determined. Each bar represents the mean of three independent experiments. Error bars, where visible, indicate the average deviation. *, *p*≤0.01 significance calculated by a Student's *t*-test. (A) The impacts of *resA* mutant and *rseA/rpoE* double mutant on the promoter activity of *rpoE* gene. (B) The impacts of *smeIJK* mutant and culture media on the promoter activity of *rpoE* gene.

Envelope stress responses (ESRs) are well documented in bacteria, with the σ^E^ being a key regulator of ESRs in several bacteria such as *E. coli* [15], *Pseudomonas aeruginosa* [16], and *Xanthomonas campestris* [17]. Because *ΔsmeIJK* may cause a compromise in the envelope integrity (Fig. 3), whether *ΔsmeIJK-*mediated ESR triggers *rpoE* regulon activation in *S. maltophilia* is of great importance. Based on the conserved genes organization of *rpoE* and anti-*rpoE* from various bacterial species, the putative *rpoE*/anti-*rpoE* operon was genome-widely searched in *S. maltophilia* K279a genome. Three flanking genes (Smlt3555-Smlt3554-Smlt3553), which encode putative σ^E^, RseA, and MucD proteins, attracted our attention (Fig. S2). The genomic organization of *rpoE-rseA-mucD* in *S. maltophilia* is identical to that in *X. campestris* pv. campestris [17], and their encoded proteins share the identities of 89%, 48%, and 66% for σ^E^, RseA, and MucD, respectively (Fig. S2). The consensus DNA sequences recognized by σ^E^ of *X. campestris* are -35 (5′-GAACTT-3′) and -10 (5′-TCTCA-3′) [18], which are identified upstream of *S. maltophilia rpoE* gene (Fig. S2), indicating that the *rpoE-rseA-mucD* operon can be a member of σ^E^ regulon in *S. maltophilia*, like that in *X. campestris* [17]. To test it, a *P_rpoE_-xylE* transcription-fusion reporter plasmid pRpoE_xylE_ was constructed. The plasmid was introduced into the wild-type KJ, KJΔRseA (an *rseA* isogenic mutant) and KJΔRpoEΔRseA (an *rpoE* and *rseA* double mutant), respectively. The *P_rpoE_* activity obviously increased when *rseA* was inactivated, and significantly decreased to the level below the wild-type when *rseA* and *rpoE* were simultaneously inactivated (Fig. 4A), indicating that the σ^E^ is a member of σ^E^ regulon in *S. maltophilia*, which has been commonly seen in many other bacteria [17]. Therefore, the extent of *rpoE* expression can be used as the indicator of σ^E^ regulon activation.

In view of the involvement of SmeIJK pump in the maintenance of envelope integrity, we further tested whether *smeIJK* inactivation activates the σ^E^ regulon. The *rpoE* expression in KJ and KJΔSmeIJK cells grown in different culture media was determined. The culture media tested included the LB, the LB without NaCl, the LB with casein hydrolysate, and the MH. Compared with wild-type, *smeIJK* mutant caused a 3.0-, 3.1-, 3.4-, and 3.7-fold increment in the *P_rpoE_*-driven C23O activity in the LB, the LB without NaCl, the LB with casein hydrolysate, and the MH, respectively. (Fig. 4B).

### MDA-mediated, σ^E^-dependent SmeIJK up-regulation

**Figure 5:**
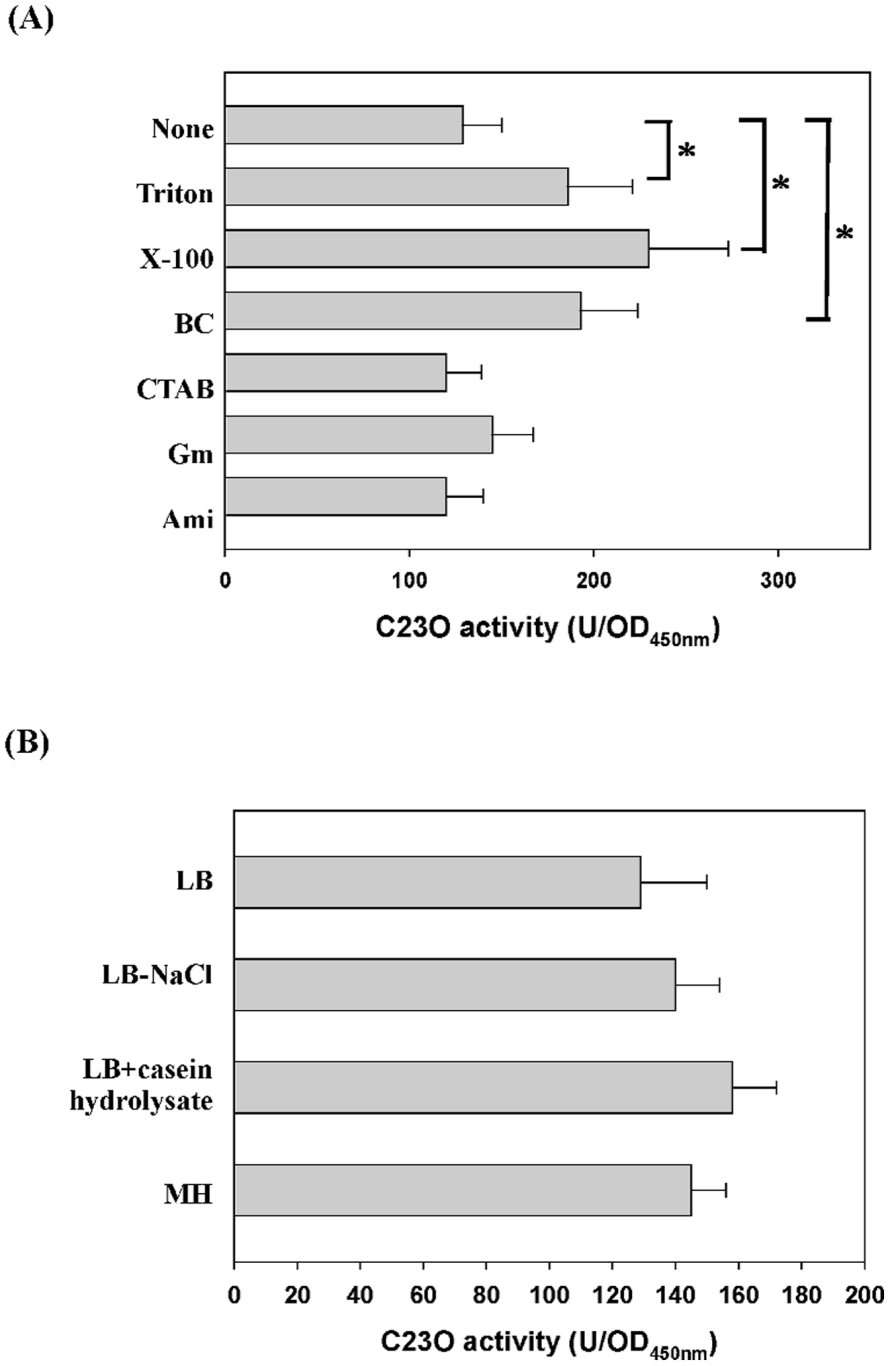
The expression of *smeIJK* operon in different stresses and culture media. Plasmid containing a transcriptional fusion of the upstream region of *smeI* to the *xylE* gene (pSmeI_xylE_) was transferred into the wild-type KJ. The C23O activities of the logarithmic-phase cultures of KJ(pSmeI_xylE_) were determined. Each bar represents the mean of three independent experiments. Each bar represents the mean of three independent experiments. *, *p*≤0.01 significance calculated by a Student's *t*-test. (A) The expression of *smeIJK* operon in different stresses. The concentrations of the stressors added were: Triton X-100, 100 µg/ml; benzalkonium chloride (BC), 10 µg/ml; cetyltributylammonium bromide (CTAB), 10 µg/ml; gentamicin (Gm), 1 µg/ml; amikacin (Ami), 1 µg/ml; and leucomycin (Leu), 0.5 µg/ml. (B) The expression of *smeIJK* operon in different culture media, including the LB, the LB without NaCl, the LB with casein hydrolysate, and the MH.

Studies of stresses that alter *smeIJK* operon expression can give important insights into its physiological function beyond antibiotics extrusion. For this purpose, a *P_smeIJK_-xylE* transcription fusion construct, pSmeI_xylE_, was prepared. The impact of stresses on the *smeIJK* expression was evaluated by monitoring the C23O activities of KJ(pSmeI_xylE_). The addition of membrane-damaging agents (MDAs) (Triton X-100) and surfactants (benzalkonium chloride (BC) and cetyltributylammonium bromide (CTAB)) moderately increased the expression of *smeIJK* operon (Fig. 5A). However, no significant increase in the C23O activity was seen when KJ(SmeI_xylE_) cells were treated with gentamicin, amikacin, and leucomycin, which are the known substrates of SmeIJK pump (Fig. 5A). Furthermore, the *smeIJK* expression of KJ cells was less relevant to the types of culture media tested (Fig. 5B)

**Figure 6:**
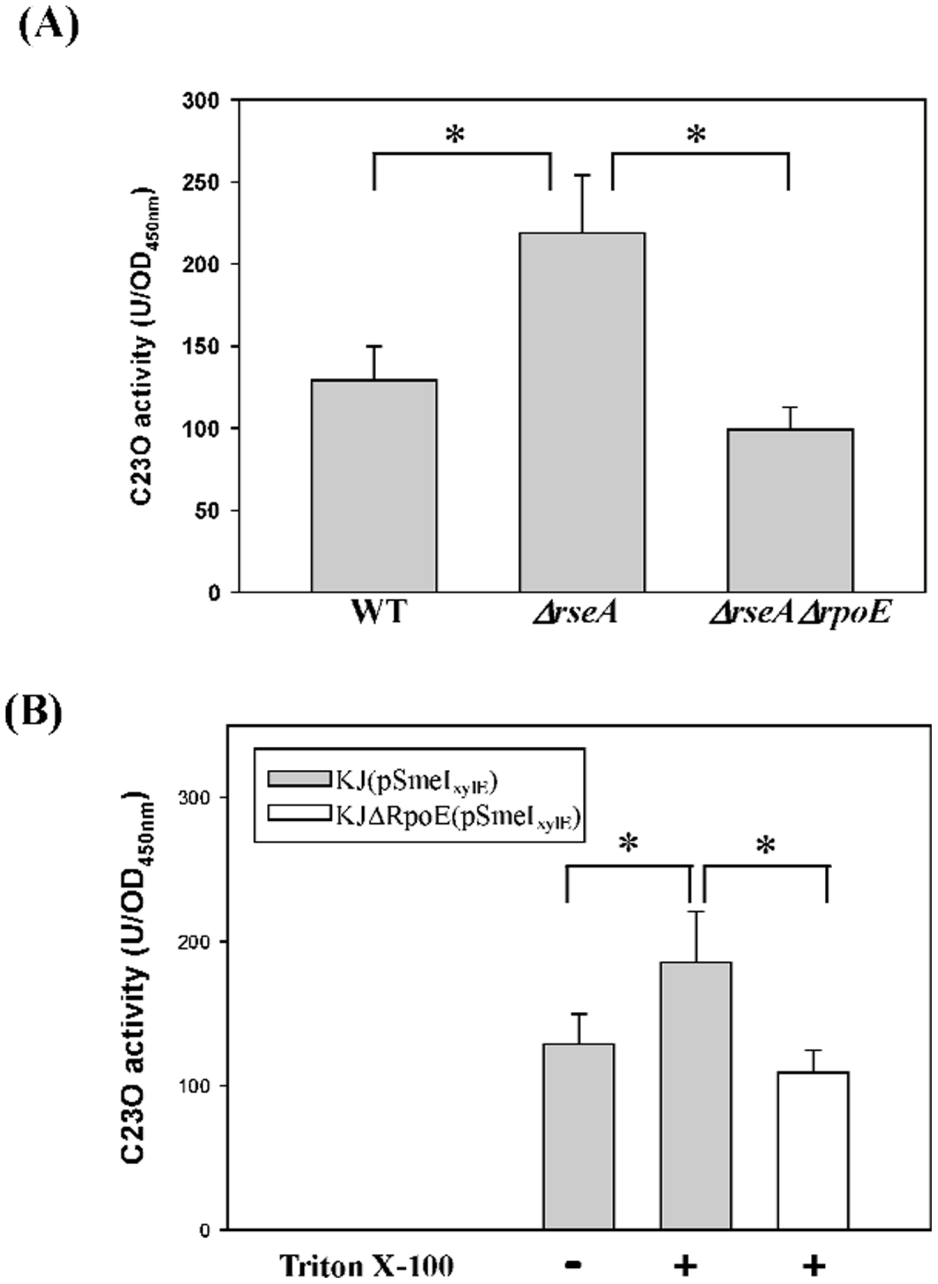
Determination of *P_smeI_* activities in different strains. Plasmid containing a transcriptional fusion of the upstream region of *smeI* to the *xylE* gene (pSmeI_xylE_) was transferred into the wild-type KJ and its derived mutants. The C23O activities of the logarithmic-phase cultures of these strains were determined. Each bar represents the mean of three independent experiments. Error bars, where visible, indicate the average deviation. *, *p*≤0.01 significance calculated by a Student's *t*-test. (A) The impacts of *resA* mutant and *rseA/rpoE* double mutant on the promoter activity of *smeIJK* operon. (B) The impacts of MDAs treatment on the promoter activity of *smeIJK* operon in the wild-type and *rpoE* mutant.

It is well perceived that the treatment of MDAs generally causes an σ^E^-mediated envelope stress response (ESR) in Gram-negative bacteria. The involvement of σ^E^ in the MDAs-mediated *smeIJK* up-regulation is of interest. Firstly, we checked whether the *smeIJK* is subjected to the regulation of *rpoE* pathway. Compared with that in the wild-type KJ, the *P_smeIJK_* activity in the *rseA* mutant increased approximately 1.7 fold and restored to the wild-type level in the *ΔrseAΔrpoE* double mutant (Fig. 6A), supporting that *rpoE* activation leads to the up-regulation of *smeIJK* operon. In addition, the *smeIJK* expression in the σ^E^-overexpression KJ cells (KJ(pRpoE)) was assessed by qRT-PCR. The *smeK* transcript in KJ(pRpoE) cells had a 2.1-fold increment compared to that in KJ(pRK415) cells, further verifying that *smeIJK* is a member of σ^E^ regulon.

Next, the involvement of *rpoE* in MDAs-mediated *smeIJK* expression was further assessed by determining the *smeIJK* expression in the wild-type KJ and in an *rpoE* knockout mutant, KJΔRpoE. As shown in Fig. 6B, the *P_smeIJK_* activity increased upon the challenge of Triton X-100 (129±21 vs. 186±35), but returned to the level as low as the non-treated counterpart once the *rpoE* was inactivated (109±16), indicating that the Triton X-100-mediated *smeIJK* up-regulation is *rpoE* dependent.

If the *smeIJK* operon is directly subjected to the regulation of *rpoE* system, the consensus DNA sequences recognized by σ^E^ should be found upstream of *smeIJK*. Since the component of *rpoE* system in *S. maltophilia* and *X. campestris* is highly similar (Fig. S2), the consensus σ^E^-binding DNA sequence of *X. campestris* [18] was tentatively employed as a search query for the σ^E^ regulon identification in *S. maltophilia*. We found a putative σ^E^ recognized −35 (5′-GAACCT-3′) and −10 (5′-TCTTA-3′) sequences upstream of the *smeIJK* operon (Fig. S3). This raises a possibility of direct regulation of *rpoE* system on the expression of *smeIJK*.

## Discussion

The organization of *smeIJK* locus is similar to those of *mdtABC* of *E. coli* [19], [20], *sdeCDE* of *Serratia macrescens* [21], [22], and *muxABC* of *P. aeruginosa* [23] in the possession of two RND-type transporters. The identities among these homologues are summarized in Table S2. For the MuxABC pump of *P. aeruginosa*, both the two RND components are essential for its function [23]. However, the MdtABC system of *E. coli* is an example to describe that the two RND transporters encoded by the same operon may have its own role [20]. The heteromultimer RND pump, MdtABC-TolC, effluxes bile salts, SDS, and novobiocin. Homomultimer RND pump MdtAC-TolC also displays the efflux function but with a narrow substrate profile limited to bile salts; in contrast, homomultimer RND pump MdtAB-TolC totally loses the efflux function [20]. In this article, with respect to the functions in antimicrobial extrusion, growth ability in MH medium, hypo-osmolarity tolerance, and cell envelope sensitivity, both RND components are not absolutely essential for pump functions, since mutants KJΔJ and KJΔK still keep considerable functions as the wild-type KJ does (Table 1, Fig. 1, 2 & 3). However, a recent study has pointed out that the SmeJ and SmeK RND transporters of *S. maltophilia* K279a are essential to produce a functional multidrug transporter [11]. The discrepancies in the necessity of two RND transporters between isolates K279a and KJ may be due to the genetic background of the bacterial host.

In this study, we observed that *smeIJK* mutant displays growth retardation in the MH medium, but not in the LB medium. Although the real mechanism responsible for this phenotype is still unclear after several tries, at least two conclusions can be made from our results. (i) The medium-dependent accumulation of noxious compounds is not the key reason for the phenotype of *smeIJK* mutant (Fig. 1B) (ii) The casein hydrolysate in the MH medium partially contributes the logarithmic-phase growth compromise of *smeIJK* mutant in the MH medium (Fig. 2D). A recent study has shown that there is a considerable culture medium-specific variability in the Cpx-mediated ESR in *E. coli* [24]. Therefore, the impact of culture medium ingredients on the ESR activation and bacterial physiology should be more intricate than we can image.

**Figure 7:**
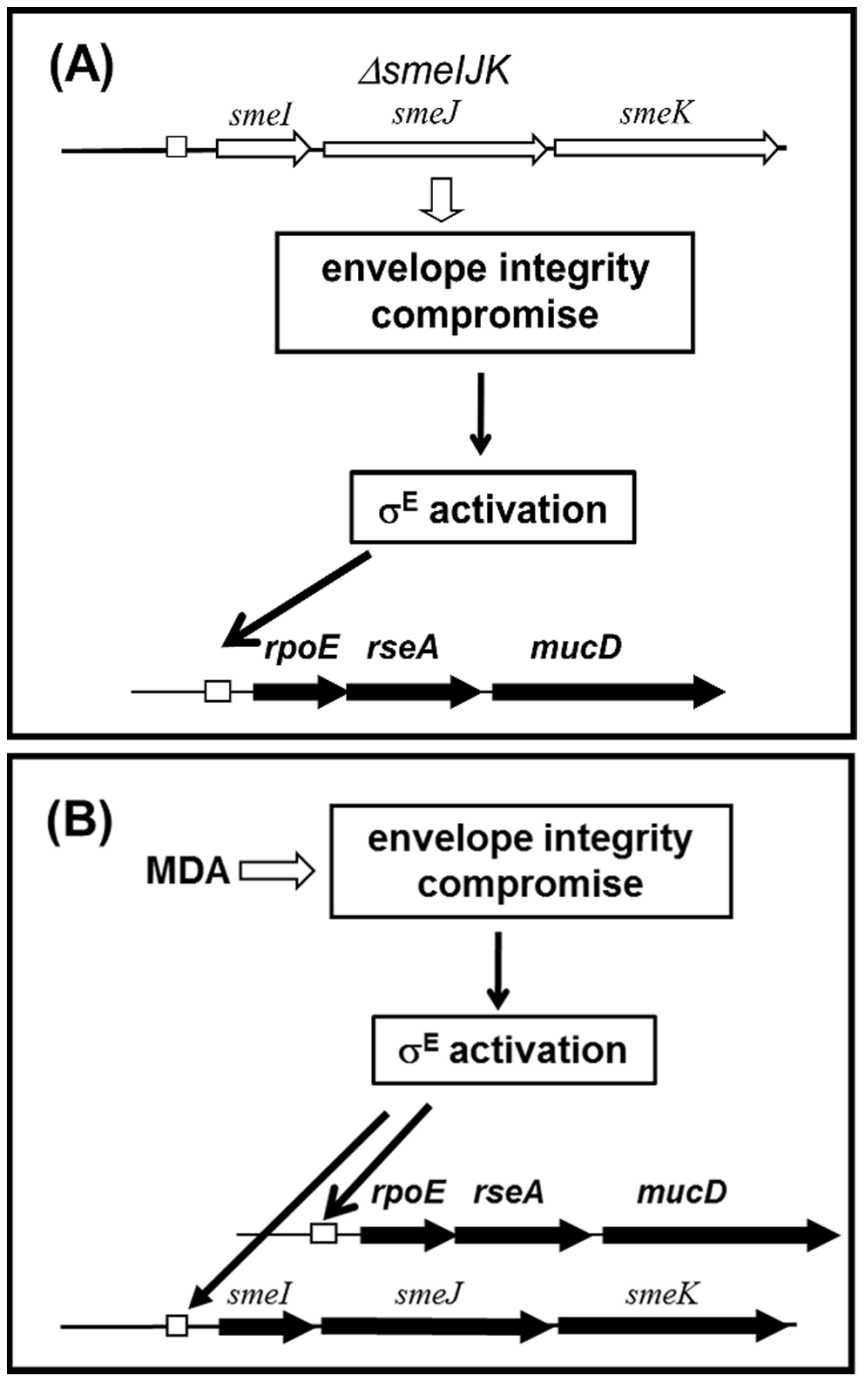
The involvement of *smeIJK* and membrane damaging agents (MDAs) in the σ^E^-mediated envelope stress response (ESR) of *S. maltophilia*. (A) Deletion of *smeIJK* compromises the cell envelope integrity and activates the *rpoE* to alleviate the envelope stress. (B) The treatment of MDAs on the wild-type cells activates *rpoE* system and upregualtes *smeIJK* expression. *SmeIJK* operon is a member of *rpoE* regulon.

For survival, bacteria harbor a variety of ESRs to deal with the envelope stresses. Six ESR systems, RpoE, CpxRA, BaeSR, Rcs-phosphorelays, phage shock protein, and vesicle release response, have been well documented in *E. coli*. Each system has unique sets of inducing stressors and downstream targets. Therefore, the ESRs are highly regulated and there are often multiple response pathways in a given microorganism. The MdtABC, a SmeIJK homologue in *E. coli*, has been proved to be a member of the BaeSR regulon; however, there is no direct evidence to support the role of MdtABC in dealing with the envelope stress [25]. In this study, we demonstrated that SmeIJK is regulated by RpoE-mediated ESR and SmeIJK has a contribution to the envelope integrity in *S. maltophilia*. In addition, EmhABC of *P. fluorescens* cLP6a [26] and MexCD-OprJ of *Pseudomonas aeruginosa* [27] are known to be involved in the ESR. The expression of *emhABC* increases when *P. florescens* is grown at 35°C (7°C up the optimum growth temperature), signifying the role of EmhABC in the management of membrane stress caused by an unfavorable incubation temperature [26]. The MexCD-OprJ efflux system appears to be a component of an ESR in *P. aeruginosa* because it is induced by a variety of MDAs in an *algU*-dependent manner [27]. It is worth mentioning that the EmhABC and *mexCD-oprJ* are constitutively weakly expressed. However, the SmeIJK pump proposed in this study is intrinsically expressed even in the absence of any antibiotics stress. Loss of the constitutively expressed SmeIJK pump may disturb the envelope stability and cause the σ^E^-mediated envelope stress (Fig. 7A). Furthermore, *smeIJK* can be further upexpressed upon the challenge of MDAs or surfactants. As illustrated in Fig. 7B, treatment with MDAs triggers the σ^E^ regulon and, thus, activates an array of genes to alleviate the envelope stresses. The *rpoE-resA-mucD* and *smeIJK* operons are the members of σ^E^ regulon. The up-regulation of the SmeIJK efflux pump may be a means by which the σ^E^ mediates adaptation to the envelope stress. The disinfectants, such as BC and CTAB, are extensively used in hospital. The physiological role of SmeIJK pump may benefit the survival of *S. maltophilia* against the disinfectants and envelope stress challenges. But, this outcome unfortunately confers the increased resistance of *S. maltophilia* to aminoglycosides and leucomycin.

## Supporting Information

Figure S1 **Schematic organization of the** ***smeIJK*** **operon and its derived mutants of** ***S. maltophilia*** **.** The *smeIJK* operon contains genes for a membrane fusion protein (*smeI*) and two RND transporters (*smeJ* and *smeK*). The orientation of gene is indicated by the arrow. The solid lines, labeled as I to IV, represent the PCR amplicons for the construction of recombinant plasmids. The numbers in the brackets represent the PCR amplicon size (bps). The white box indicates the deleted region for each mutant construct.
(DOCX)

Figure S2 **Schematic organization of the** ***rpoE-rseA-mucD cluster*** **, its derived mutants and the predicted σ^E^ binding site upstream of the** ***rpoE*** **in** ***S. maltophilia*** **.** The orientation of gene is indicated by the arrow. The white box indicates the deleted region. The each protein identity of the *rpoE* region between *X. campestris* pv. *campestris* and *S. maltophilia* is indicated. The gray lines, labeled as I to III, represent the PCR amlpicons for the construction of recombinant plasmids. The numbers in the brackets represent the PCR amplicon size (bps). The sequence of the putative *rpoE* promoter region is shown below the map. The putative −35/−10 regions are underlined, based on the reported consensus sequence for the σ^E^-regulated promoter elements of *X. campestris* pv. campestris.
(DOCX)

Figure S3 **The DNA sequences upstream of the** ***smeIJK*** **operon.** The orientation of gene is indicated by the arrow. The putative −35/−10 regions of the *rpoE* promoter are boxed, based on the reported consensus sequence for the σ^E^-regulated promoter elements of *X. campestris* pv.campestris.
(DOCX)

Table S1 **Bacterial strains, plasmids and primers used in this study.**
(DOCX)

Table S2 **The homologues of SmeIJK efflux pump.**
(DOCX)
