# Supplementary material for: A Linkage between SmeIJK Efflux Pump, Cell Envelope Integrity, and σE-Mediated Envelope Stress Response in Stenotrophomonas maltophilia
Source: PLoS One. 2014 Nov 12;9(11):e111784. doi: 10.1371/journal.pone.0111784 (PMC4229105; doi:10.1371/journal.pone.0111784)
Supplement: Figure S1 — Schematic organization of the smeIJK operon and its derived mutants of S. maltophilia . The smeIJK operon contains genes for a membrane fusion protein (smeI) and two RND transporters (smeJ and smeK). The orientation of gene is indicated by the arrow. The solid lines, labeled as I to IV, represent the PCR amplicons for the construction of recombinant plasmids. The numbers in the brackets represent the PCR amplicon size (bps). The white box indicates the deleted region for each mutant construct. (DOCX) [file pone.0111784.s001.docx]

*smeI*

*smeJ*

*smeK*

KJ

KJΔJ

KJΔK

KJΔJK

KJΔIJK

II(578)

III(589)

IV(519)

I(465)

**Fig. S1. Schematic organization of the *smeIJK* operon and its derived mutants of *S. maltophilia*.** The *smeIJK* operon contains genes for a membrane fusion protein (*smeI*) and two RND transporters (*smeJ* and *smeK*). The orientation of gene is indicated by the arrow. The solid lines, labeled as I to IV, represent the PCR amplicons for the construction of recombinant plasmids. The numbers in the brackets represent the PCR amplicon size (bps). The white box indicates the deleted region for each mutant construct.
