# Supplementary material for: A Linkage between SmeIJK Efflux Pump, Cell Envelope Integrity, and σE-Mediated Envelope Stress Response in Stenotrophomonas maltophilia
Source: PLoS One. 2014 Nov 12;9(11):e111784. doi: 10.1371/journal.pone.0111784 (PMC4229105; doi:10.1371/journal.pone.0111784)
Supplement: Figure S2 — Schematic organization of the rpoE-rseA-mucD cluster , its derived mutants and the predicted σE binding site upstream of the rpoE in S. maltophilia . The orientation of gene is indicated by the arrow. The white box indicates the deleted region. The each protein identity of the rpoE region between X. campestris pv. campestris and S. maltophilia is indicated. The gray lines, labeled as I to III, represent the PCR amlpicons for the construction of recombinant plasmids. The numbers in the brackets represent the PCR amplicon size (bps). The sequence of the putative rpoE promoter region is shown below the map. The putative −35/−10 regions are underlined, based on the reported consensus sequence for the σE-regulated promoter elements of X. campestris pv. campestris. (DOCX) [file pone.0111784.s002.docx]

*rseA*

*mucD*

*rpoE*

***X. campestris***

**pv. *campestris***

89% 48% 66%

Protein identity (%)

*rpoE*

(Smlt3555)

*rseA*

(Smlt3554)

*mucD*

(Smlt3553)

***S. maltophilia***

**K279a**

I(414) II(346) III(385)

**KJΔRpoE**

**KJΔRseA**

**KJΔRpoEΔRseA**

5’-GGCGACT**GAACTT**TTCCCGGGATTGGCAG**TCTCA**TTGCCCGACGTCGGTTGGGCTGACAGGAGTGCGGCCCCTC**ATG**GCC -3’

*rpoE*

**Fig. S2. Schematic organization of the *rpoE-rseA-mucD cluster*, its derived mutants and the predicted σ^E^ binding site upstream of the *rpoE* in *S. maltophilia*.** The orientation of gene is indicated by the arrow. The white box indicates the deleted region. The each protein identity of the *rpoE* region between *X. campestris* pv. *campestris* and *S. maltophilia* is indicated. The gray lines, labeled as I to III, represent the PCR amlpicons for the construction of recombinant plasmids. The numbers in the brackets represent the PCR amplicon size (bps). The sequence of the putative *rpoE* promoter region is shown below the map. The putative -35/-10 regions are underlined, based on the reported consensus sequence for the σ^E^-regulated promoter elements of *X. campestris* pv. campestris.
