# Supplementary material for: A Linkage between SmeIJK Efflux Pump, Cell Envelope Integrity, and σE-Mediated Envelope Stress Response in Stenotrophomonas maltophilia
Source: PLoS One. 2014 Nov 12;9(11):e111784. doi: 10.1371/journal.pone.0111784 (PMC4229105; doi:10.1371/journal.pone.0111784)
Supplement: Figure S3 — The DNA sequences upstream of the smeIJK operon. The orientation of gene is indicated by the arrow. The putative −35/−10 regions of the rpoE promoter are boxed, based on the reported consensus sequence for the σE-regulated promoter elements of X. campestris pv.campestris. (DOCX) [file pone.0111784.s003.docx]

*smeI*

*smeJ*

*smeK*

5’-ccgccacgggaacctccgcggccggatccggtcttagcgcccgtcccatccgtcatgcaggaaacgtgatgtggcacaggtatgctttgcgcattgccaaccggggccgcgccccgtccagccctcttggagtcctcgta**ATG-**3’

***smeI***

**Fig. S3. The DNA sequences upstream of the *smeIJK* operon.** The orientation of gene is indicated by the arrow. The putative -35/-10 regions of the *rpoE* promoter are boxed, based on the reported consensus sequence for the σ^E^-regulated promoter elements of *X. campestris* pv.campestris.
