# Supplementary material for: A Linkage between SmeIJK Efflux Pump, Cell Envelope Integrity, and σE-Mediated Envelope Stress Response in Stenotrophomonas maltophilia
Source: PLoS One. 2014 Nov 12;9(11):e111784. doi: 10.1371/journal.pone.0111784 (PMC4229105; doi:10.1371/journal.pone.0111784)
Supplement: Table S1 — Bacterial strains, plasmids and primers used in this study. (DOCX) [file pone.0111784.s004.docx]

**Table S1 Bacterial strains, plasmids and primers used in this study**

| **Strain, plasmid, or primer** | **Genotype or properties** |
| --- | --- |
| ***S. maltophilia*** |  |
| KJ | Wild type, a clinical isolate from Taiwan |
| KJΔJ | *S. maltophilia* KJ mutant of *smeJ* gene; *ΔsmeJ* |
| KJΔK  KJΔJK | *S. maltophilia* KJ mutant of *smeK* gene; *ΔsmeK*  *S. maltophilia* KJ mutant of *smeJ* and *smeK* genes; *ΔsmeJ ΔsmeK* |
| KJΔIJK | *S. maltophilia* KJ mutant of *smeIJK* operon; *ΔsmeIJK* |
| KJΔRpoE | *S. maltophilia* KJ mutant of*rpoE*gene; *ΔrpoE* |
| KJΔRseA | *S. maltophilia* KJ mutant of *rseA*gene; *ΔresA* |
| KJΔRpoEΔRseA | *S. maltophilia* KJ mutant of *rpoE*and*rseA* genes; *ΔrpoE, ΔrseA* |
|  |  |
| ***Escherichia coli*** |  |
| DH5α | F- φ80d*lacZ*Δ*M15* Δ(*lacZYA-argF*)*U169 deoR recA1 endA1hsdR17* (r_k_^-^m_k_^+^) *phoA supE44λ^-^thi-1 gyrA96 relA1* |
| S17-1  Plasmids | λpir + mating strain |
| pEX18Tc | *sacBoriT*, Tc^r^ |
| pRK415 | Derived from pRK415, replacing the tetracycline resistance gene with kanamycin resistance gene; Km^r^ |
| pTXylE  pRKXylE | Plasmid containing the *xylE* cassette; Amp^r^  A pRK415-derived vector for the construction of promoter-*xylE* transcription fusion, the orientation of *xylE* gene in this plasmid is opposite to that of *P_lacZ_* of pRK415; Tc^r^ |
| pΔSmeJ | pEX18Tc vector with a 1167-bp DNA fragment of *S. maltophilia* KJ, containing the *smeJ* gene with an internal 1903-bp deletion; Tc^r^ |
| pΔSmeK | pEX18Tc vector with a 1108-bp DNA fragment of *S. maltophilia* KJ, containing the *smeK* gene with an internal 2571-bp deletion; Tc^r^ |
| pΔSmeIJK | pEX18Tc vector with a 984-bp DNA fragment of *S. maltophilia* KJ, containing the smeIJK operon with an internal 7180-bp deletion; Tc^r^ |
| pΔRpoE  pΔRseA | pEX18Tc vector with a 760-bp DNA fragment of *S. maltophilia* KJ, containing the *rpoE* gene with an internal 230-bp deletion; Tc^r^  pEX18Tc vector with a 731-bp DNA fragment of *S. maltophilia* KJ, containing the *resA* gene with an internal 545-bp deletion; Tc^r^ |
| pSmeI_xylE_ | pRK415 with a 413-bp DNA fragment upstream from the *smeI*start codon and a *P_smeRo_::xylE* transcriptional fusion |
| pRpoE_xylE_ | pRK415 with a 322-bp DNA fragment upstream from the *rpoE*start codon and a *P_rpoE_::xylE* transcriptional fusion |
| pRpoE | pRK415 with an intact *rpoE* gene, Tc^R^ |
| Primers  SmeI5-F  SmeI5-R  SmeJ5-F  SmeJ5-R  SmeK5-F  SmeK5-R  SmeK3-F  SmeK3-R  RpoE5-F  RpoE5-R  RpoE3-F  RpoE3-R  RseA3-F  RseA3-R  SmeKQ-F  SmeKQ-R  rDNA-F  rDNA-R | 5’-CTTCGAGCTCAAGCCCATCGCAC-3’  5’-CCGGTACCAGAACCGCCACGAC-3’  5’-ATCAAGCTTTTCTCGATGGACCGCG -3’  5’-GCGACGGAATTCCAGCAGCACC -3’  5’-ACCAGATCTCGATCATCTCCACGC-3’  5’-TAGGAATTCAGGCCGACCATGAAC-3’  5’-GGTAAGCTTCCTGTCCACGCTG-3’  5’-ATCTGCAGTTCTACGCACGCTG-3’  5’-GCGAATTCAGGGAGAGGACCACCA-3’  5’-TTGGGTACCAGGACATCGAACGC-3’  5’-GCGGTACCGAACAGTTCGACAGT-3’  5’-GCTCTAGATGGTTCTGCGATTCGT-3’  5’-GCCAAGCTTACCCGCAGCCAGCAG-3’  5’-GGGGTACCATCGGACGGCAACCT-3’  5’-AACTCCGACCCCAGCGAC-3  5-GCGATCATCGAGATCACCGAC-3’  5’- GACCTTGCGCGATTGAATG -3’  5’- CGGATCGTCGCCTTGGT -3’ |
