# Supplementary material for: A Linkage between SmeIJK Efflux Pump, Cell Envelope Integrity, and σE-Mediated Envelope Stress Response in Stenotrophomonas maltophilia
Source: PLoS One. 2014 Nov 12;9(11):e111784. doi: 10.1371/journal.pone.0111784 (PMC4229105; doi:10.1371/journal.pone.0111784)
Supplement: Table S2 — The homologues of SmeIJK efflux pump. (DOCX) [file pone.0111784.s005.docx]

**Table S2 The homologues of SmeIJK efflux pump**

| Protein homologues | Bacterium | Protein identities to SmeI/SmeJ/SmeK (%) |
| --- | --- | --- |
| MdtA/MdtBMdtC  SdeC/SdeD/SdeE  MuxA/MuxB/MuxC | *Escherichia coli*  *Serratiamacrescens*  *Pseudomonas aeruginosa* | 42 / 50 / 44  45 / 50 / 43  42 / 52 / 45 |
